# Body composition and associated factors among 5–7‐year‐old children with moderate acute malnutrition in Jimma town in southwest Ethiopia: A comparative cross‐sectional study

**DOI:** 10.1111/mcn.13655

**Published:** 2024-04-25

**Authors:** Melese Sinaga Teshome, Tamirat Bekele, Evi Verbecque, Sarah Mingels, Marita Granitzer, Teklu Gemechu Abessa, Tefera Belachew Lema, Eugene Rameckers

**Affiliations:** ^1^ Department of Nutrition and Dietetics, Faculty of Public Health, Health Institute Jimma University Jimma Ethiopia; ^2^ Rehabilitation Research Centre (REVAL), Rehabilitation Sciences and Physiotherapy Hasselt University Diepenbeek Belgium; ^3^ Department of Nutrition and Dietetics, Faculty of Public Health Jimma University Jimma Ethiopia; ^4^ Musculoskeletal Research Unit, Department of Rehabilitation Sciences, Faculty of Kinesiology and Rehabilitation Sciences Leuven University Leuven Belgium; ^5^ Department of Special Needs and Inclusive Education Jimma University Jimma Ethiopia; ^6^ Research School CAPHRI, Department of Rehabilitation Medicine Maastricht University Maastricht The Netherlands; ^7^ Centre of Expertise in Rehabilitation and Audiology Hoensbroek The Netherlands

**Keywords:** body composition, children, Ethiopia, fat‐free mass, fat mass, MAM, pre‐school

## Abstract

Acute malnutrition affects not only the growth and development but also the body composition of children. However, its specific effects have not yet been characterized. This study aims to compare the body composition of 5–7‐year‐old children with moderate acute malnutrition (MAM) to that of their well‐nourished (WN) peers and identify associated factors. A school‐based comparative cross‐sectional study was conducted from June to July 2022 in Jimma town, southwest Ethiopia. The study participants were selected from eight kindergartens and eight primary schools using a simple random sampling technique based on the proportional allocation of the sample to the size of the population in the respective school. Descriptive statistics and multivariable linear regression analyses were used to assess the mean differences and associations between variables and isolate independent predictors of body composition, respectively. The statistical significance was determined using ß‐coefficients with 95% confidence intervals and a *p* value of ≤ 0.05. Data were captured from 388 (194 MAM and 194 WN) children with a response rate of 97.9%. The mean fat‐free mass of WN children was significantly higher compared with those with MAM (*p* < 0.001). The mean (SD) of fat mass of MAM children was 4.23 ± 0.72 kg, 4.36 ± 0.88 kg and 4.08 ± 0.89 kg for 5, 6 and 7‐year‐olds, respectively. For WN children, the mean (SD) of fat mass was 4.92 ± 0.88 kg for 5 years old, 5.64 ± 1.01 kg for 6 years old and 5.75 ± 1.26 kg for 7 years old (*p* < 0.001). On the multivariable linear regression analysis after controlling for background variables, WN children exhibited 1.51 times higher fat‐free mass compared with MAM children (*β* = 1.51, *p* = 0.003). A unit increase in age of the study participants was associated with a 1.37 increment in fat‐free mass (*β* = 1.37, *p * < 0.001). WN children had 1.07 times higher fat mass compared with children with MAM (*β* = 1.07, *p* < 0.001). A unit increase in the age of the child resulted in 0.15 times increment in fat mass (*β* = 0.15, *p* = 0.020), and being female was associated with a 0.37 increase in fat mass (*β* = 0.37, *p* < 0.001). The results showed that the mean fat mass and fat‐free mass were significantly lower among moderately acute malnourished children than in WN children showing the loss of both body compartments due to malnutrition. The body mass index for age, age of the child and sex of the child were significantly linked to both fat‐free mass and fat mass.

## Key messages

- This study demonstrated that children aged 5–7 years suffering from moderate acute malnutrition (MAM) have lower mean body fat mass (FM) and fat‐free mass (FFM) as compared with well‐nourished (WN) children.
- The substantial difference observed between MAM and WN children in body FM and FFM calls for the integration of MAM treatment into the routine health services to prevent their sliding down to SAM and associated mortality.

## Graphical abstract

Our results showed that the mean fat mass (FM) and fat‐free mass (FFM) were significantly lower among moderate acute malnutrition (MAM) children than among NW children, showing the effect of moderate thinness on wasting of muscle mass, which can help to improve the continuum of care in the management of acute malnutrition.

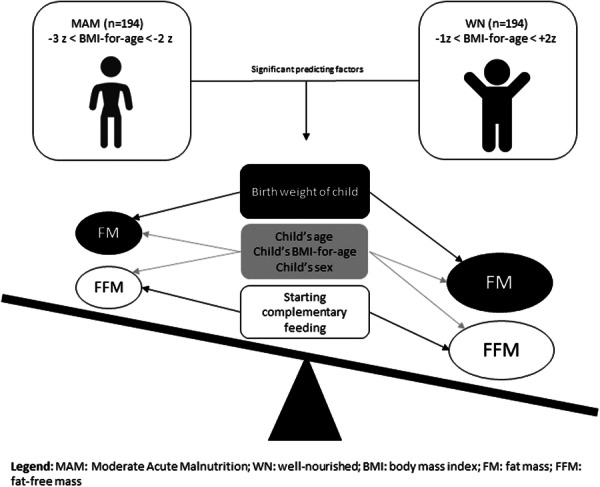

## 1 INTRODUCTION

According to the World Health Organization (WHO), a child is well‐nourished (WN) if their body mass index (BMI) for their age is between −2 and +1 and has moderate acute malnutrition (MAM) if their BMI is greater than −3 and less than −2 (Cashin & Oot, 2018). Malnutrition is broadly categorized as undernutrition (which includes wasting [low weight‐for‐height], underweight [low weight‐for‐age], stunting [low height‐for‐age], and micronutrient‐related malnutrition), and overnutrition (Dukhi, 2020; Kamruzzaman et al., 2021; World Health Organization, 2017).

Acute malnutrition (AM) is a significant global public health concern, with a more acute impact in low‐ and middle‐income countries (LMICs). Nearly 64% of people with AM are diagnosed with MAM, which is more common than severe acute malnutrition (SAM) (Karim et al., 2021).

Malnutrition has been linked to stunting, wasting and underweight conditions that are characterized by altered body composition and decreased physical growth (Wells, 2019). The muscle may be killed in favour of general organ protection, yet shrinkage of the thymus brought on by undernutrition has been linked to decreased immunological capability (Prentice, 1999). Fat mass (FM) supplies immune system function with substantial metabolic costs, in terms of metabolic precursors and energy (Wells, 2010). Leptin which acts as a ‘gateway’ for immunological activity is also secreted by FM (Lord, 2002). Malnutrition has a detrimental effect on the physical, intellectual, social and emotional development of children (Park et al., 2001). Evidence shows that malnutrition causes weak muscles and a decrease in motor skills in children (Badaru et al., 2023, Renault & Quesada, 1993).

Understanding the effect of wasting on fat‐free mass (FFM) and FM might substantiate public health and therapeutic interventions that are more successful in promoting survival, long‐term quality of life and decreasing functional consequences (Sinha et al., 2022). The basic body composition model divides body mass into two components, the FFM and FM, to better understand the physical impacts of malnutrition (Wells, 2019). Body composition is a potentially useful indicator as it is crucial for both long‐ and short‐term survival in both sick and healthy children (Radhakrishna et al., 2010). The body adiposity proportion is a useful predictor of children's health and nutritional status since they change with age, sex and environmental factors (Debnath et al., 2018). Adipocytes are found in numerous other cells and organs throughout the body (Gallagher, 2020).

Previous studies have shown the drawbacks of using body mass index for these objectives since it does not differentiate between increases in FFM and FM. Additionally, too much FM can conceal deficits in FFM (Kyle et al., 2015). Exclusive traditional weight measurement (one‐compartment model) does not provide as much information on nutritional storage and the type of tissue accumulation as a body composition assessment (McDonald et al., 2019).

Currently, anthropometric measurements based on middle upper arm circumference (MUAC), height, weight, weight for length z‐score or both, are used to assess and treat children with MAM, but neither FFM nor FM is used (Bartz et al., 2014). Evidence shows that body composition during childhood may predict the risk of developing a noncommunicable disease (NCD) during adulthood (Bander et al., 2023). Especially, rapid or catch‐up weight gain during early childhood has been linked to insulin resistance, overweight or obesity, adiposity and NCDs in later life (Kristjansson et al., 2015; Wells, 2019).

Children and adolescents aged 5–19 have heightened nutritional requirements due to their rapid growth, and undernutrition can lead to poor growth, delayed sexual maturation and impaired cognitive development. Furthermore, overweight and obesity during this age range are likely to persist into adulthood and increase the risk of chronic diseases. Therefore, assessing the nutritional status of this age group is crucial for further management and interventions (Agency for International Development [USAID], 2018). In Ethiopia, the treatment of MAM is not provided as a routine service except in some food‐insecure areas, like the Integrated Management of Acute Malnutrition (IMAM) woredas. It is believed that the health extension programme will take care of MAM cases (James et al., 2016). However, children suffering from MAM and with no access to supplementary feeding programmes experience high rates of deterioration and no improvement, as evidenced by a prospective cohort study conducted in rural Ethiopia (James et al., 2016).

Brain development continues for an extended period post‐natally. The brain increases in size fourfold during the pre‐school period, reaching approximately 90% of adult volume by age 6 (Lenroot & Giedd, 2006; Stiles & Jernigan, 2010). According to research by Luciana and Nelson, in typically developing children between the ages of 4 and 8, the prefrontal working memory system develops at about age 4 and gets better between the ages of 5 and 7 years (Luciana & Nelson, 1998), and there is a significant improvement in the ability to store visual short‐term memory. In diverse cultures, 5–7 years of age is the beginning of the ‘age of reason’ (Rogoff et al., 1975). The age range of 5–7 years old is a sensitive period/golden age, when the highest level of activity throughout life happens.

Nevertheless, there is a paucity of evidence on body composition and associated factors among children in this age group in Ethiopia. Therefore, this study aimed to compare the body composition of WN and moderately wasted children aged 5–7 years in Jimma town, southwest Ethiopia.

## 2 METHODS AND MATERIALS

### 2.1 Study area and period

The study was carried out in kindergartens (KG) and elementary schools within Jimma Town, which is located 345 km from Addis Ababa, the capital of Ethiopia. The geographical coordinates of the town are approximately 7°41′N latitude and 36°50′E longitude. The town is bordered to the east by Kersa Woreda, to the west by Mana Woreda, to the north by Mana and Kersa Woreda, and the south by Seka Woreda. Data obtained from Jimma Town municipality show that the town has a total surface area of 4623 hectares and a population of 224,565 of which 39,352 children under the age of 5. Of the total of under 5 years old children, 19,645 were pre‐schoolers spread across 17 Kebeles. There were 25 public elementary schools in the Town. A total of 32,443 students were in public primary schools; of those, 10,123 were grade 1 students and 8770 were KG students in the age group of 5–7 years. Twenty‐five KG classes are primary schools called ‘0’ classes locally.

A school‐based comparative cross‐sectional study was conducted from June to July 2022 among 5–7‐year‐old children with MAM and WN children at eight KG and primary schools. Eligible participants were 5–7‐year‐old children attending KG and primary schools in Jimma Town, and whose parents or caregivers are residents of the town and willing to participate. Exclusions comprised mothers or caregivers who were seriously ill, children who were seriously ill, and physically disabled and children diagnosed with MAM and on an outpatient therapeutic programme (OTP). For children diagnosed with MAM, nutrition counselling was provided to their mothers or caregivers, and for those diagnosed with SAM, a linkage was established with a nearby health facility for further treatments.

### 2.2 Sample size and sampling techniques

The sample size was determined using G*power statistical software version 3.1.9.4 with the assumption of a difference between two independent samples (*t* test) for comparing the mean. Input parameters were: effect size of 0.4, a precision of 0.05, power (1‐β) of 80%, with a 95% level of confidence, and an allocation ratio of 1:1 which resulted in a sample size of 200. A design effect of 1.8 was applied for the cluster sampling, and 10% was added for nonresponse, resulting in a total sample size of 396. So, the study required the involvement of 198 WN and 198 MAM children.

### 2.3 Sampling procedure

In each of the 25 public primary schools in Jimma town, there are KG classes (locally called ‘0’ classes). The study's sampling process involved two steps. First, eight primary schools were randomly selected using a lottery method using the WHO recommendation as a rule of thumb to include at least 30% of the total schools (WHO & Unicef, 2009). Second, students were categorized into two groups based on age: KG (age group 5–6 years) and grade 1 (age group 7 years). For the age group of 5–7 years, the sample size was allocated based on the proportion of the age group that the school has, which is one‐third (*n* = 132) of the sample from grade 1 (7‐year‐old) children in the primary school and two‐third (*n* = 264) of the sample size from KG classes (5–6‐year‐old) children. The registration book of the school was used to select the study units from eligible children. Finally, a simple random sampling technique was applied to select the final sample after screening the children for their nutritional status as either MAM or WN.

### 2.4 Data collection and procedure

Data were collected using a semistructured questionnaire through face‐to‐face interviews of variables including socioeconomic and demographic factors, household food security (access) and child health characteristics. The wealth index of the household was assessed using ownership of durable assets. The wealth index was ranked into poor, medium and rich socioeconomic status tertiles. One physiotherapist, three nurses and two nutritionists who are fluent in the local language participated as data collectors and supervisors, respectively. Data collectors received 2‐day training on the questionnaire and data collection procedures. Throughout the data collection process, both the supervisors and the principal investigator followed the data collectors and performed quality checks.

### 2.5 Household food insecurity access scale measurement

Household food insecurity was measured using the Household Food Insecurity Access Scale (HFIAS), which was developed by the Food and Nutrition Technical Assistance (FANTA) project. For HFIAS measurement, each of the questions was asked with a recall period of 4 weeks. The respondent was first asked an occurrence question, that is, whether the condition in the question happened at all in the past 4 weeks (yes or no). If the respondent answered ‘yes’ to an occurrence question, a frequency‐of‐occurrence question was asked to determine whether the condition happened rarely (once or twice), sometimes (three to 10 times), or often (more than 10 times) in the past 4 weeks (Coates et al., 2007; Gebreyesus et al., 2015).

### 2.6 Anthropometric measurements

The measurements of height (cm) and weight (kg) were taken for each child after the standardization of measures and calibration of equipment. All measurements were taken three times, and an average value was recorded. Weight was measured with shoes taken off and in light clothing (underwear and t‐shirts only) using a Seca digital weighing scale (Seca, Model 770) and recorded to the nearest 0.1 kg. Height was measured using a stadiometer Seca 213 (Seca) recorded to the nearest 0.1 cm, after positioning the subject in a Frankfurt plane. In measuring height, the head was positioned in the Frankfort and the ankle, calf, buttocks and shoulders touched the vertical stand of the stadiometer.

### 2.7 Body composition measurement

Body composition (FFM and FM) was measured using bioelectrical impendence analysis on the right side of the body. The International Society for the Advancement of Kinanthropometry's recommendations for anatomical landmarks were followed (Kamruzzaman et al., 2021). Before beginning the body composition measurement, the study participants were asked to remove tight hand and wrist jewellery since it might interfere with the bioelectrical impedance analysis (BIA) body composition instrument. During BIA measurement, the study participants were asked to lie in a supine position on a flat table with the arms comfortably abducted from the body at 15° and the legs separated. The arms were separated from the trunk of the body and the legs were not touching each other (Health & Survey, 2000). Measurements were performed in duplicate at all points, and an average was taken. Bioelectrical impedance measurement was conducted by standard equipment, which is Bodystat QuadScan 4000.

### 2.8 Data quality management

Before actual data collection, a pretest was conducted on 20 subjects at a nearby school (Swiss‐Ginjo school) not included in the study. All the anthropometric measurements were performed by both the investigator and trained (data collector) three nurses, two nutritionists and supervisors to eliminate within‐examiner error. The weight scale was calibrated to zero level with no object on it and placed on a leveled surface before the measurement was performed. A continuous checkup of scales and bioelectrical impendence was carried out for their reliability. The data collection was supervised by the principal investigator and trained supervisors. The principal investigator supervised and reviewed every questionnaire for completeness and logical consistency and made corrections on the spot. A standardization exercise was conducted during training to capture technical errors in measurement (TEM). The TEM was performed to assess interobserver and interobserver errors using a published methodology (Perini et al., 2005) and the coefficient of variation was checked.

### 2.9 Data analysis

The data were checked for completeness, coded and entered EPI Data Version 3.1 and exported to SPSS Version 26 for cleaning and analysis. Principal component analysis (PCA) was done for the household wealth index. Household food security status was computed based on the HFIAS nine questions of occurrence and frequency of occurrence. The data were analysed based on food insecurity access classification criteria and categorized as a food‐secure or food‐insecure household. We performed a *χ* ^2^ test for the categorical data and an independent sample *t* test was used to compare differences in the mean value of body composition between MAM and WN children.

Descriptive statistics (mean ± SD, frequencies and percentages) and bivariate analysis were utilized to select candidate variables for multivariable analysis. To determine the relationship between body composition and explanatory variables, a simple linear regression was conducted. Before the analysis, several assumptions were checked, including the linearity between the dependent and independent variables, normality, independence of observations (no multicollinearity) and homoscedasticity. The bivariate linear regression identified variables with *p* values less than 0.25 which were then considered for multivariable linear regression. This was done to determine the factors that independently affect body composition. During the analysis, all interactions were checked. The results were presented using ß‐coefficients and 95% confidence intervals. A *p* value less than 0.05 was considered statistically significant and the model's fitness was checked using adjusted *R* ^2^.

#### 2.9.1 Operational definitions

1. Food insecurity: ‘A situation that exists when people lack secure access to sufficient amounts of safe and nutritious food for normal growth and development and an active and healthy life’ (Coates et al., 2007).
2. MAM: a child with BMI‐for‐age of ≥−3 to <−2 z scores (USAID, 2018).
3. WN: a child with BMI‐for‐age ≥ −2 to < −1 z scores (USAID, 2018).
4. FM: That portion of the human body that is composed strictly of fat (Health & Survey, 2000).

### 2.10 Ethics statement

The Institutional Review Board of Jimma University Institute of Health has ethically approved the proposal. Additionally, the administrative education office of Jimma Town received a letter of support from the Department of Human Nutrition and Dietetics, which this office then disseminated to each selected school. Each child's mother provided written informed consent. For children diagnosed with MAM mothers or caregivers received nutritional counselling, and those diagnosed with severe acute malnutrition were linked to local health facilities.

## 3 RESULTS

### 3.1 Sociodemographic characteristics of the mothers and caregivers

The response rate was 97.9%, with 388 of the 396 children and mothers/primary caregivers providing complete responses. These results were confirmed by the values of the technical error of measurement for weight and height in the acceptable range. For weight intra and interobserver variation was 0.11 kg which is acceptable (<0.21 kg), whereas for height it was 0.65 cm which is acceptable (<1.0 cm). The coefficient of variation was also 2.2% for height and 1.8% for weight which is within the acceptable range of less than 3%. The mean age of study the participants was 32.08 (±7.57) years. A total of 79.6% of the households were food insecure (Table 1).

**Table 1:** Sociodemographic characteristics of parents/caregivers of children in Jimma town southwest Ethiopia, June to July 2022 (*n* = 388).

Variables	Category	Nutritional status of the child (*n* = 388)			
		MAM (*n* = 194)	WN (*n* = 194)	Total	
		*n* = 194 (%)	*n* = 194 (%)	*n* = 388 (%)	*p* Value
Age (years) of mother/caregiver	≤19	7 (3.6)	0	7 (1.8)	<0.0001
	20–29	54 (27.8)	87 (44.8)	141 (36.3)	
	30–39	98 (50.5)	88 (45.4)	186 (47.9)	
	≥40	35 (18.0)	19 (9.8)	54 (13.9)	
Age (years) of mother/caregiver Mean (±SD)		32.08 (±7.57)			
Marital status of the caregiver	Married & live together	109 (56.2)	168 (86.6)	277 (71.4)	<0.0001
	Married & and live separately	21 (10.8)	5 (2.6)	26 (6.7)	
	Divorced	27 (13.9)	14 (7.2)	41 (10.6)	
	Widowed	29 (14.9)	6 (3.1)	35 (9)	
	Single	8 (4.1)	1 (0.5)	9 (2.3)	
Educational status of the mother	Cannot read and write	110 (56.7)	39 (20.1)	149 (38.4)	<0.0001
	Can read and write	24 (12.4)	32 (16.5)	56 (14.4)	
	Primary school (0–8)	47 (24.2)	64 (33)	111 (28.6)	
	Secondary school (9–12)	11 (5.7)	55 (28.4)	66 (17)	
	Above secondary school ( >12)	2 (1)	4 (2.1)	6 (1.5)	
The educational status of the father	Cannot read and write	41 (31.5)	11 (6.4)	52 (17.1)	<0.0001
	Can read and write	13 (10)	10 (5.8)	23 (7.6)	
	Primary school (0–8)	34 (26.2)	50 (28.9)	84 (27.7)	
	Secondary school (9–12)	32 (24.6)	64 (37)	96 (31.7)	
	Above secondary school ( >12)	10 (7.7)	38 (22)	48 (15.8)	
Occupation of mother	Housewife	80 (41.2)	68 (35.1)	148 (38.1)	<0.0001
	Merchant	23 (11.9)	32 (16.5)	55 (14.2)	
	Government employee	6 (3.1)	41 (21.1)	47 (12.1)	
	Private‐employee	20 (10.3)	27 (13.9)	47 (12.1)	
	Daily labourer	65 (33.5)	26 (13.4)	91 (23.5)	
Occupation of father	Farmer	3 (2.3)	4 (2.3)	7 (2.3)	<0.0001
	Merchant	14 (10.8)	52 (30)	66 (21.8)	
	Government employee	30 (23.1)	73 (42.2)	103 (34)	
	Private‐employee	24 (18.5)	12 (6.9)	36 (11.9)	
	Daily labourer	54 (41.5)	29 (16.8)	83 (27.4)	
	Other^a^	5 (3.8)	3 (1.7)	8 (2.6)	
Ethnicity	Oromo	134 (69.1)	129 (66.5)	263 (67.8)	0.218
	Amhara	16 (8.2)	23 (11.9)	39 (10.1)	
	Kafa	7 (3.6)	13 (6.7)	20 (5.2)	
	Yem	20 (10.3)	11 (5.7)	31 (8)	
	Other^b^	17 (8.8)	18 (9.3)	35 (9)	
Religion	Muslim	103 (53.1)	100 (51.5)	203 (52.3)	0.971
	Orthodox	55 (28.4)	58 (29.9)	113 (29.1)	
	Protestant	35 (18)	35 (18)	70 (18)	
	Other^c^	1 (0.5)	1 (0.5)	2 (0.5)	
Family size	≤5 members	140 (72.2)	162 (83.5)	302 (77.8)	0.007
	>5 members	54 (27.8)	32 (16.5)	86 (22.2)	
Head of household	Father	133 (68.6)	174 (89.7)	307 (79.1)	<0.0001
	Mother	61 (31.4)	20 (10.3)	81 (20.9)	
Household wealth index	Poor	136 (70.1)	48 (24.7)	184 (47.4)	<0.0001
	Medium	34 (17.5)	39 (20.1)	73 (18.8)	
	Rich	24 (12.4)	107 (55.2)	131 (33.8)	
Food security status	Food secured	9 (4.6)	70 (36)	79 (20.4)	<0.0001
	Food insecure	185 (95.4)	124 (64)	309 (79.6)	

*Note*: Significant at *p* < 0.05.

^a^ Other: drivers, temporary employees, cajoler& retired.

^b^ Other: Silte, Dawro, and Tigre.

^c^ Other: Adventist and catholic.

### 3.2 Sociodemographic characteristics of the study participants' children

Out of 388 participants, 212 (54.6%) were females. The mean age of these participants was 6.08 (±0.76). A large (44.1%) proportion was in KG‐3. The majority (92.3%) of the participants travel to school by foot. The mean distance from the living place to the school amounts to 15.13 (±6.38) minutes (Table 2).

**Table 2:** Sociodemographic characteristics of children aged between 5–7 years in Jimma town southwest Ethiopia, June to July 2022 (*n* = 388).

Variables	Category	Nutritional status of the child (*n* = 388)			
		MAM (*n* = 194)	WN (*n* = 194)	Total	
		*n* = 194 (%)	*n* = 194 (%)	*n* = 388 (%)	*p* Value
Sex	Male	78 (40.2)	98 (50.5)	176 (45.4)	0.041
	Female	116 (59.8)	96 (49.5)	212 (54.6)	
Age of child (years)	5	55 (28.4)	43 (22.2)	98 (25.3)	0.308
	6	75 (38.7)	87 (44.8)	162 (41.8)	
	7	64 (33)	64 (33)	128 (32.9)	
Grade Level	KG2	67 (34.5)	53 (27.3)	120 (30.9)	0.307
	KG3	81 (41.8)	90 (46.4)	171 (44.1)	
	Grade 1	46 (23.7)	51 (26.3)	97 (25.0)	
Distance from the school (minutes)	<10	24 (12.4)	16 (8.2)	40 (10.3)	0.003
	10‐14	53 (27.3)	30 (15.5)	83 (21.4)	
	≥15	117 (60.3)	148 (76.3)	265 (68.3)	
Distance from the school (minutes)^a^ Mean ( ± SD)			15.13 (±6.38)		
Means of transportation	Foot	183 (94.3)	175 (90.7)	358 (92.3)	0.177
	Taxi	11 (5.7)	19 (9.3)	30 (7.7)	
Time to start CF^b^	Before 6 months	147 (75.8)	53 (27.3)	200 (51.5)	<0.001
	At 6 months	36 (18.6)	65 (33.5)	101 (26.0)	
	After 6 months	11 (5.7)	76 (39.2)	87 (22.4)	

*Note*: Significant at *p* < 0.05.

^a^ Indicate that this is based on the estimated parental report.

^b^ CF: complementary feeding time as reported by the mother.

### 3.3 Comparison of body composition

There were significant differences between MAM and WN children in body composition. In comparison to MAM children of 5, 6 and 7 years of age, WN children had a significantly larger FM (*p* < 0.001) (Table 3).

**Table 3:** Comparison of FM and FFM per age category between WN and MAM children of age 5–7 years in Jimma town southwest Ethiopia, June to July 2022.

Outcomes variables	Age (years)	BMI for age category	*N*	Mean	Std	Difference		*p* ^a^
						Mean	(95% CI)	
Fat mass (kg)	5	MAM	55	4.23	0.72	−0.69	−1.02, −0.36	<0.001
		Well‐nourished	43	4.92	0.88			
	6	MAM	75	4.36	0.88	−1.28	−1.57, −0.98	<0.001
		Well‐nourished	87	5.64	1.01			
	7	MAM	64	4.08	0.89	−1.67	−2.05, −1.28	<0.001
		Well‐nourished	64	5.75	1.26			
Fat‐free mass (kg)	5	MAM	55	11.26	1.24	−0.68	−1.26, −0.10	0.022
		Well‐nourished	43	11.95	1.57			
	6	MAM	75	12.38	1.73	−0.88	−2.20, 0.42	0.184
		Well‐nourished	87	13.27	5.89			
	7	MAM	64	13.46	1.83	−2.03	−3.02, −1.04	<0.001
		Well‐nourished	64	15.49	3.53			

^a^ *p* value deducted from the independent sample *t* test.

There were significant differences between MAM and WN children in body fat percentage, dry lean mass, basal metabolic rate, body FM index and FFM index (*p* < 0.001) (Table 4).

**Table 4:** Independent sample t‐test of body composition children of age 5–7 years in Jimma town southwest Ethiopia, June to July 2022.

Body composition	BMI for age category	*N*	Mean	Std	Difference		*p* *
					Mean	(95% CI)	
Body fat percentage	MAM	194	25.28	4.798	−4.590	−5.61, −3.56	<0.001
	Well‐nourished	194	29.87	5.436			
Fat mass (kg)	MAM	194	4.23	0.849	−1.288	−1.48, −1.08	<0.001
	Well‐nourished	194	5.52	1.121			
Lean mass (%)	MAM	194	74.19	6.835	4.412	3.02, 5.79	<0.001
	Well‐nourished	194	69.78	7.032			
Dry lean mass (kg)	MAM	194	2.80	0.491	−0.323	−0.49, −0.15	<0.001
	Well‐nourished	194	3.13	1.082			
Fat‐free mass (kg)	MAM	194	12.42	1.852	−1.291	−2.00, −0.57	<0.001
	Well‐nourished	194	13.71	4.686			
Total body water (%)	MAM	194	56.20	5.036	1.793	0.44, 3.14	0.010
	Well‐nourished	194	54.40	8.149			
Total body water (lt)	MAM	194	9.61	3.386	−0.659	−1.26, −0.05	0.032
	Well‐nourished	194	10.27	2.605			
Body cell mass	MAM	194	9.78	2.818	−1.890	−2.61, −1.16	<0.001
	Well‐nourished	194	11.67	4.286			
Intracellular water (%)	MAM	194	41.58	8.350	−1.110	−2.80, 0.58	0.199
	Well‐nourished	194	42.69	8.658			
Intracellular water in volume	MAM	194	7.17	3.329	−0.592	−1.13, −0.04	0.033
	Well‐nourished	194	7.76	1.945			
Extracellular water (%)	MAM	194	44.00	5.732	2.429	1.27, 3.58	<0.001
	Well‐nourished	194	41.57	5.881			
Extracellular water in volume	MAM	194	7.65	4.998	−0.164	−1.02, 0.69	0.708
	Well‐nourished	194	7.82	3.516			
Basal metabolic rate (Kcal)	MAM	194	877.13	39.40	−37.00	−45.5, −28.4	<0.001
	Well‐nourished	194	914.14	45.71			
Basal metabolic rate/body weight (Kcal/kg)	MAM	194	52.51	3.665	2.525	1.59, 3.45	<0.001
	Well‐nourished	194	49.99	5.494			
Body fat mass index	MAM	194	3.38	0.724	−1.001	−1.16, −0.83	<0.001
	Well‐nourished	194	4.38	0.931			
Fat‐free mass index	MAM	194	9.18	0.814	−1.036	−1.32, −0.74	<0.001
	Well‐nourished	194	10.22	1.871			

Abbreviations: BMI, body mass index; MAM, moderate acute malnutrition.

* *p* value deducted from the independent sample *t* test.

### 3.4 Predictors of body composition (FFM and FM)

The following variables had a *p* value less than 0.25 for the bivariate linear regression for the FFM BMI for age, age of mother/caregiver, sex of the child, age of the child, head of the house, complimentary food starts after 6 months, school absenteeism on days, the child's immunization status, distance from the school in minutes, history of current illness, household food insecurity, maternal education and wealth index were candidate variables for the multivariable linear regression analysis (Table A1).

The following variables in bivariate linear regression had a *p* value of less than 0.25 for FM: BMI for age, child's sex, age and birthweight, head of household, complementary food started before 6 months, complementary food started after 6 months, child's exclusive breastfeeding (EBF) for the first 6 months, child's immunization status, history of current illness, household food insecurity, maternal education, and wealth index. These variables were candidate  for the multivariable linear regression analysis (Table A2).

### 3.5 Independent predictors of FFM

On multivariable linear regression analyses, after adjusting for other variables, BMI for age, sex of the child, age of the child and starting complementary feeding after 6 months were significantly associated with FFM. WN children have 1.51 times higher FFM than MAM children (*β* = 1.51, *p* = 0.003). An increase in the child's age by 1 year is associated with a 1.37‐fold increment in FFM (*β* = 1.37, *p* < 0.001). Being female was significantly associated with a decrease in FFM by 0.76 (*β* = −0.76, *p* = 0.03). Starting complementary feeding after 6 months was significantly negatively associated with FFM (*β* = −1.06, *p* = 0.023) (Table 5).

**Table 5:** Multivariable linear regression model predicting fat‐free mass among children 5–7 years old in Jimma town southwest Ethiopia, June to July 2022.

Model	Unstandardized Coefficients		*p*	95% CI	
	*B*	Std. Err		Lower	Upper
BMI for age (without MAM)	1.511	0.504	0.003	0.520	2.502
Age of mother/caregiver	−0.041	0.023	0.074	−0.087	0.004
Maternal education (literate)	0.454	0.612	0.459	−0.750	1.657
Age of the child (years)	1.374	0.229	<0.001	0.924	1.823
Sex of the child (female)	−0.763	0.349	0.030	−1.450	−0.076
After 6‐month starting complementary feeding	−1.067	0.467	0.023	−1.986	−0.147
Head of the house (mother)	−0.468	0.662	0.480	−1.770	0.835
School absenteeism on days	−0.032	0.033	0.334	−0.096	0.033
Distance from the school (minutes)	−0.025	0.028	0.359	−0.080	0.029
Immunization status of the child	0.010	0.494	0.985	−0.963	0.982
History of current illness	−0.349	0.461	0.449	−1.255	0.556
Food insecurity (secure)	−0.039	0.494	0.937	−1.010	0.933
Wealth index (rich)	0.115	0.443	0.796	−0.756	0.986

*Note*: Maximum VIF = 2.361.

Abbreviations: BMI, body mass index; MAM, moderate acute malnutrition.

### 3.6 Independent predictors of FM

On the multivariable linear regression model, after adjusting for other variables, BMI for age, sex of the child, age of the child and birthweight of the child were significant predictors of FM. WN children have 1.07 times higher FM than MAM children (*β* = 1.07, *p* < 0.001). A unit increase in the age of the child leads to a 0.15‐fold increment of the FM (*β* = 0.15, *p* = 0.020). Being female significantly increased the FM by 0.37 (*β* = 0.37, *p* < 0.001). The FM increases 0.37 times (*β* = 0.37, *p* = 0.012) if the birthweight of the children increases by 1 g (Table 6).

**Table 6:** Multivariable linear regression model predicting fat mass among children 5–7 years old in Jimma town southwest Ethiopia, June to July 2022.

Model	Unstandardized Coefficients		P	95% CI	
	B	Std. Err		Lower	Upper
BMI for age (without MAM)	1.072	0.145	<0.001	0.787	1.357
Maternal education (literate)	−0.084	0.122	0.492	−0.324	0.156
Age of the child (years)	0.155	0.066	0.020	0.025	0.286
Sex of the child (female)	0.378	0.102	<0.001	0.179	0.578
Birthweight of the child (gram)	0.376	0.148	0.012	0.085	0.667
Before 6‐month starting complementary feeding	0.137	0.239	0.568	−0.334	0.607
After 6‐month starting complementary feeding	0.072	0.149	0.630	−0.221	0.365
Immunization status of the child	0.263	0.149	0.077	−0.029	0.555
Child EBF for the first 6 months	−0.178	0.234	0.448	−0.638	0.283
History of current illness	0.049	0.136	0.717	−0.218	0.317
Dewormed in last 6 months	0.019	0.119	0.872	−0.216	0.254
Food insecurity (secure)	−0.253	0.144	0.081	−0.537	0.031
Wealth index (rich)	0.000	0.130	0.998	−0.256	0.256

*Note*: Maximum VIF = 5.896.

Abbreviations: BMI, body mass index; EBF, early breastfeeding; MAM, moderate acute malnutrition.

On the multivariable linear regression model, after adjusting for other variables, BMI for age, sex, age and birthweight of the child were found to be significant predictors of FM. WN children exhibit 1.07 times higher FM than MAM children (*β* = 1.07, *p* < 0.001). A 1‐year increase in the age of the child leads to a 0.15‐fold increment of the FM (*β* = 0.15, *p* = 0.020). Being female was associated significantly with an increase in FM by 0.38 (*β* = 0.376, *p* < 0.001). For each gram increase in birthweight of the children, FM increases by 0.37 times (Table 6).

## 4 DISCUSSION

This study aimed to compare body FM and FFM between 5 and 7‐year‐old children with MAM and WN counterparts. The results showed that there is a substantial difference between MAM and WN children in both body FM and FFM. This means that MAM children exhibited less FM and FFM than WN children, which is consistent with the report of a study in India (Sinha et al., 2022). Evidence suggests that MAM or wasting is associated with notable deficiencies in FFM and FM and that these deficits could last for a long time (Wells, 2016) and undernutrition does, in fact, negatively affect muscle mass, which is linked to functional deficiencies, according to scientific records (Wells, 2019).

It was also observed that MAM children's mean FM is lower than that of WN children which is not consistent with the report of the study conducted in India (Savanur & Ghugre, 2016). The difference might be due to study setting, sociodemographic and socioeconomic factors, nutritional awareness, knowledge and culture affecting nutritional status among school‐age children.

Starting complementary feeding after 6 months was negatively associated with FFM (*p* = 0.023), which is inconsistent with a study conducted in Rotterdam, Netherlands (Van Beijsterveldt et al., 2021). The possible explanations might be that breastfeeding is sufficient in fulfilling all nutrients only up to six and hence not initiating complementary feeding at 6 months may lead to loss of the FFM. It was also observed that a monthly increase in the age of the study participants led to a 1.37‐fold increase in the FFM (*p* < 0.001) and a 0.15‐fold increase in the FM (*p* = 0.020). This finding is consistent with the reports of studies conducted in Rotterdam, the Netherlands, Sweden and Minnesota Masonic (Forsum et al., 2019; Scheurer et al., 2017; Van Beijsterveldt et al., 2021). Given the presence of a high prevalence of wasting among children in Ethiopia, the findings have wider practical implications for the identification and management of children with wasting and for correcting abnormalities of body composition across all age groups.

For the paediatric clinical and research field, bioimpedance analysis (BIA) is a relatively easy‐to‐use, fast and noninvasive technique (Wang & Hui, 2015). Unlike the BIA, the BMI has failed to appropriately represent changes in body composition evaluation such as changes in body FM (Zhao et al., 2023). Lack of muscle mass is linked to several issues, including weakened muscles, lowered immunity, and an increased chance of infection (Mastorci et al., 2017). Body composition assessment can be used to identify the body compartment affected by malnutrition because of its ease of use, affordability, safety and noninvasive nature, which will have an implication on the health of children. Children go through a lot of physical changes from the pre‐school years until puberty, including linear growth and gains in muscle and fat. The method of BIA is recognized for estimating low muscle mass (Gonzalez et al., 2018). To distinguish moderately acute malnourished children from nonmalnourished ones, it is recommended that future research focuses on developing body composition cut‐off points using larger sample sizes. A comprehensive understanding of the relationship between clinical outcomes, nutritional status and body composition in children with MAM and WN ones requires further research. In Ethiopia, the management of children with MAM is only carried out in a few IMAM woredas (James et al., 2016). This leaves children with MAM in other woredas untreated, assuming that the health extension programme will address their needs. However, there has been a recent effort to integrate the treatment of MAM into routine health care. The findings suggest that there is a need to strengthen the fast implementation of these efforts to reduce the negative consequences of MAM on children.

### 4.1 Limitations and strengths of the study

The fact that body composition was assessed by using Bioelectrical Bodystat QuadScan 4000 yields objective information on body composition characteristics, including FM and FFM, which is the strength of the study.

It is important to note that there is a chance that respondents may have answered questions in a way that they think is socially desirable, which could affect the accuracy of the results. Nevertheless, steps were taken to minimize the impact of this bias by informing respondents that their responses were only being used for comparison purposes and would not affect their privacy or service use.

## 5 CONCLUSION

The study found that children aged 5–7 with MAM have lower mean FM and FFM compared with WN children. These results highlight the negative impact of MAM and emphasize the need for improved efforts to manage AM. The child's BMI for age, age and sex were significantly associated with the FFM and FM of the children. Further research is required in Ethiopia to establish cut‐off points for normal and malnourished children's body composition, using larger sample sizes.

## AUTHOR CONTRIBUTIONS

Melese Sinaga Teshome, Tefera Belachew Lema, and Eugene Rameckers conceptualized and designed the work. Data acquisition, analysis, interpretation, collection, drafting, and reviewing the paper were performed by Melese Sinaga Teshome, Tamirat Bekele, Evi Verbecque, Sarah Mingels, MaritaGranitzer, Teklu Gemechu Abessa, Tefera Belachew Lema and Eugene Rameckers. Melese Sinaga Teshome, who is the corresponding author, drafted the manuscript and submitted it for publication. All authors reviewed and approved the final version of the manuscript for publication.

## CONFLICT OF INTEREST STATEMENT

The authors declare no conflict of interest.

## Data Availability

The corresponding author will provide the datasets used and analysed during the current work upon reasonable request.

## APPENDIX A

**Table A1:** Bivariate analysis showing the candidate variables in the study fat‐free mass of children aged between 5 and 7 years in Jimma town southwest Ethiopia, June to July 2022.

Predictors	Unstandardized coefficients		*p*	95% CI for B	
	ß	Std. Err		Lower	Upper
BMI for age (without MAM)	1.291	0.362	<0.001	0.580	2.003
Age of mother/caregiver	−0.043	0.024	0.076	−0.091	0.005
Family size of the caregiver	−0.079	0.099	0.430	−0.274	0.117
Maternal education (literate)	−0.604	0.406	0.138	−1.401	0.194
Paternal education (literate)	0.077	0.378	0.839	−0.666	0.820
Age of the child (years)	1.465	0.230	<0.001	1.012	1.918
Sex of the child (female)	−1.136	0.365	0.002	−1.854	−0.419
Birthweight of the child (gram)	0.446	0.428	0.298	−0.395	1.287
Complementary food starts before 6 months	0.367	0.367	0.319	−0.356	1.089
Complementary food starts after 6 months	−0.532	0.440	0.228	−1.397	0.333
Head of the house (mother)	−0.897	0.450	0.047	−1.782	−0.012
School absenteeism on days	−0.095	0.029	0.001	−0.153	−0.038
Distance from the school in minutes	−0.041	0.029	0.153	−0.098	0.015
Immunization status of the child	−0.567	0.486	0.244	−1.521	0.388
Child EBF for the first 6 months	0.411	0.367	0.263	−0.311	1.133
History of current illness	0.575	0.374	0.125	−0.160	1.310
Dewormed in last 6 months	0.144	0.388	0.710	−0.618	0.907
Food insecurity (secure)	−0.642	0.455	0.160	−1.537	0.254
Wealth index (medium)	−0.414	0.450	0.359	−1.298	0.471
Wealth index (rich)	−0.599	0.368	0.105	−1.323	0.125

Abbreviations: BMI, body mass index; EBF, exclusive breastfeeding; MAM, moderate acute malnutrition.

**Table A2:** Bivariate analysis showing the candidate variables in the study fat mass of children aged between 5 and 7 years in Jimma town southwest Ethiopia, June to July 2022.

Predictors	Unstandardized coefficients		*p*	95% CI for B	
	*ß*	Std. Err		Lower	Upper
BMI for age (without MAM)	1.288	0.101	<0.001	1.09	1.48
Age of mother/caregiver	−0.002	0.008	0.763	−0.01	0.01
Family size of the caregiver	−0.013	0.033	0.685	−0.07	0.05
Maternal education (literate)	−0.606	0.130	<0.001	−0.86	−0.35
Age of the child (years)	0.174	0.079	0.028	0.01	0.32
Sex of the child (female)	0.192	0.121	0.113	−0.04	0.42
Birthweight of the child (gram)	0.922	0.132	<0.001	0.66	1.18
Complementary food starts before 6 months	−0.666	0.116	<0.001	−0.89	−0.43
Complementary food starts after 6 months	0.631	0.141	<0.001	0.35	0.90
Immunization status of the child	−0.490	0.157	0.002	−0.79	−0.18
Child EBF for the first 6 months	−0.707	0.115	<0.001	−0.93	−0.48
History of current illness	0.887	0.114	<0.001	0.66	1.11
Dewormed in last 6 months	−0.448	0.125	<0.001	−0.69	−0.20
Food insecurity (secure)	−0.912	0.142	<0.001	−1.19	−0.63
Wealth index (medium)	−0.013	0.147	0.931	−0.30	0.27
Wealth index (rich)	−0.791	0.114	<0.001	−1.01	−0.56

Abbreviations: BMI, body mass index; EBF, exclusive breastfeeding; MAM, moderate acute malnutrition.
